# Tackling Dementia Together via The Australian Dementia Network (ADNeT): A Summary of Initiatives, Progress and Plans

**DOI:** 10.3233/JAD-230854

**Published:** 2023-11-21

**Authors:** Sharon L. Naismith, Johannes C. Michaelian, Cherry Santos, Inga Mehrani, Joanne Robertson, Kasey Wallis, Xiaoping Lin, Stephanie A. Ward, Ralph Martins, Colin L. Masters, Michael Breakspear, Susannah Ahern, Jurgen Fripp, Peter R. Schofield, Perminder S. Sachdev, Christopher C. Rowe

**Affiliations:** a Healthy Brain Ageing Program, School of Psychology, Charles Perkins Centre and the Brain and Mind Centre, University of Sydney, Sydney, New South Wales, Australia; b The University of Melbourne, Melbourne, Victoria, Australia; c Centre for Healthy Brain Ageing (CHeBA), Discipline of Psychiatry and Mental Health, University of New South Wales, Sydney, Australia; d The Florey Institute, The University of Melbourne, Parkville, Victoria, Australia; e School of Public Health and Preventive Medicine, Monash University, Melbourne, Victoria, Australia; f Department of Geriatric Medicine, The Prince of Wales Hospital, Sydney, New South Wales, Australia; g School of Medical Sciences, Edith Cowan University, Perth, Western Australia, Australia and Department of Biomedical Sciences, Macquarie University, Sydney, New South Wales, Australia; h School of Psychology, College of Engineering, Science and the Environment, University of Newcastle, New South Wales, Australia and School of Medicine and Public Health, College of Health and Medicine, University of Newcastle, New South Wales, Australia; i Australian e-Health Research Centre, Commonwealth Scientific and Industrial Research Organisation (CSIRO), Queensland, Australia; j Neuroscience Research Australia, Sydney, Australia and School of Biomedical Sciences, University of New South Wales, Sydney, Australia; k Neuropsychiatric Institute, Prince of Wales Hospital, Randwick, New South Wales, Australia; l Department of Molecular Imaging and Therapy, Austin Health, The University of Melbourne, Victoria, Australia

**Keywords:** ADNeT, Alzheimer’s disease, Australian Dementia Network, clinical quality registry, clinical trials, dementia, diagnosis, health services, memory clinic, mild cognitive impairment

## Abstract

In 2018, the Australian Dementia Network (ADNeT) was established to bring together Australia’s leading dementia researchers, people with living experience and clinicians to transform research and clinical care in the field. To address dementia diagnosis, treatment, and care, ADNeT has established three core initiatives: the *Clinical Quality Registry* (CQR), *Memory Clinics*, and *Screening for Trials*. Collectively, the initiatives have developed an integrated clinical and research community, driving practice excellence in this field, leading to novel innovations in diagnostics, clinical care, professional development, quality and harmonization of healthcare, clinical trials, and translation of research into practice. Australia now has a national Registry for Mild Cognitive Impairment and dementia with 55 participating clinical sites, an extensive map of memory clinic services, national Memory and Cognition Clinic Guidelines and specialized screening for trials sites in five states. This paper provides an overview of ADNeT’s achievements to date and future directions. With the increase in dementia cases expected over coming decades, and with recent advances in plasma biomarkers and amyloid lowering therapies, the nationally coordinated initiatives and partnerships ADNeT has established are critical for increased national prevention efforts, co-ordinated implementation of emerging treatments for Alzheimer’s disease, innovation of early and accurate diagnosis, driving continuous improvements in clinical care and patient outcome and access to post-diagnostic support and clinical trials. For a heterogenous disorder such as dementia, which is now the second leading cause of death in Australia following cardiovascular disease, the case for adequate investment into research and development has grown even more compelling.

## INTRODUCTION

Worldwide, there are more than 55 million persons living with dementia, with nearly 10 million new cases each year according to the World Health Organization (WHO) [1]. In 2019, the estimated total global societal cost of dementia was estimated at US$1.3 trillion, and these costs are expected to surpass US$2.8 trillion by 2030 as both the number of people living with dementia and care costs increase [1]. In Australia, more than 400,000 people across a total population of approximately 26 million are living with dementia, and with an aging population this is projected to increase to more than a million people by 2058 [2]. Based on the Australian Institute of Health and Wellbeing (AIHW) report on dementia, one in 12 Australians over the age of 65 has dementia. It is the second leading cause of death among Australians and the leading cause of death for women [2]. Over half of the people living in permanent residential aged care have dementia [2]. The economic and personal costs of dementia are staggering. In 2018, dementia was estimated to have cost Australia more than AU$15 billion. By 2025, the total cost of dementia is predicted to increase to AU$18.7 billion in today’s dollars, and by 2056, to more than AU$36.8 billion [3].

Pertinent to addressing the health care burden, costs, and impact of dementia, is the need to consider Australia’s health system preparedness for timely and optimized diagnosis and care, and to build a pipeline for more rapid and efficient research translation. Reform is needed not only for established dementia, but also for people living with mild cognitive impairment (MCI) and preclinical Alzheimer’s disease, where rapid research advances in accurate diagnostics and pharmacological and non-pharmacological treatments are occurring.

In Australia, there is considerable heterogeneity of diagnostic services and processes for MCI and dementia within primary care [4], and within Australian Memory and Cognition Clinics and other service settings, with the reporting of little harmonization or standardization of processes [5, 6]. The application of positron emission tomography (PET) imaging of brain amyloid or cerebrospinal fluid (CSF) measures of amyloid and tau in clinical practice to confirm an underlying Alzheimer’s disease process has been very limited due to patient cost and restricted access; processes for identifying and referring individuals living with dementia into clinical trials have been inefficient; and very few clinics have had the resources or expertise to provide cognitive interventions, post-diagnostic support or other evidence-based non-pharmacological interventions [6]. For MCI, while there is compelling evidence that a range of interventions can be offered [6, 7], these are rarely provided in clinical practice [5]. In contrast to other countries such as Austria, Korea, Spain, Sweden, and the United States of America [8], there has been a lack of Australian data on the quality of care and there have not been coordinated efforts to systematically monitor the quality of care with a view to improving and standardizing service provision at a national level. Finally, in comparison to other international exemplars, such as the United Kingdom Memory Services National Accreditation Programme (MSNAP) [9], Australian dementia services have lacked a clinical community for peer support, clinical care professional development, service enhancements and for statewide or national service and health system advocacy. Compounding the service issues, research funding for dementia has been historically low in comparison to other chronic diseases and relative to international efforts. Hence, there has been a need to synergize efforts, coordinate nationally, invest in infrastructure, build research workforce capacity, and support and enable translational research.

## FORMING A NATIONAL NETWORK FOR DEMENTIA RESEARCH

In July 2018, the Australian Dementia Network (ADNeT) [10] was founded as a result of a competitive grant funding process of the National Health and Medical Research Council of Australia (NHMRC). The investment of AU$18 million over 5 years represented 9% of the total NHMRC National Institute for Dementia Research (NNIDR) budget and was the single largest grant. ADNeT was created to address research fragmentation and bring together some of Australia’s leading researchers with clinicians and people with living experience, and thereby establish a powerful network for dementia prevention, treatment, and care. A further AU$2 million was provided to ADNeT by philanthropic donors, the Yulgilbar Foundation and the Wicking Trust.

**Fig. 1:**
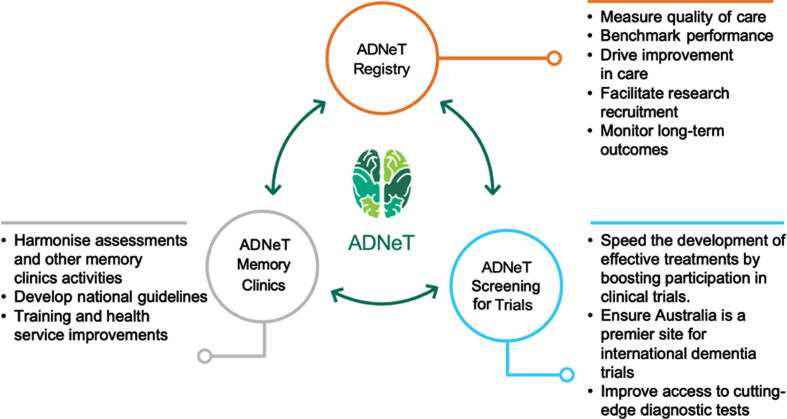
Three principal initiatives of the Australian Dementia Network (ADNeT).

Since its inception in 2018, ADNeT has built a unique national dementia community that draws together the expertise of researchers from 17 universities and research institutions across Australia (for more information, visit: https://www.australiandementianetwork.org.au/). ADNeT has established three principal national initiatives: a Clinical Quality Registry (CQR), a Memory Clinics (MCs) network addressing health services, and the Screening for Trials Initiative (STI), that facilitates the screening and enrolment of participants into clinical trials (see Fig. 1).

### The Clinical Quality Registry Initiative

Building on the work of the SveDem, the ADNeT Clinical Quality Registry (CQR) was established [11]. The ADNeT CQR is the first registry in Australia for dementia and MCI [12] and critically, it has adopted the standards required for a Clinical Quality Registry [13]. It measures the quality of diagnosis and care for people newly diagnosed with dementia or identified as having MCI. The long-term aim is to register all persons diagnosed with dementia or identified as having MCI, independent of the diagnostic setting and in doing so, systematically drive continuous improvements in the quality of care and patient outcomes.

Eligible patients for the registry are currently identified by the sites who have consented to participate, including public and private memory clinics, other dementia and MCI diagnostic services (e.g., aged care outreach services), and individual medical practitioners involved in the diagnosis of dementia and MCI (e.g., geriatricians, neurologists and psychiatrists). A unique recruitment framework, comprising of an opt-out approach and waivers of consent, is used to overcome challenges in recruiting people with cognitive impairments and to maximize coverage [14].

The ADNeT CQR collects data from participating sites and, where appropriate, registry participants and their carers. Data collected from participating sites are based on a baseline minimum dataset which includes patient demographics (e.g., age and sex), and clinical information (e.g., diagnosis, dementia risk factors, diagnostic investigations) information. Following recruitment, where appropriate, registry participants and their carers (if identified) are invited to complete a survey which includes questions on patient/carer-reported outcomes and their experience of clinical care at the point of diagnosis.

To understand long-term health outcomes and service use of registry participants, the ADNeT CQR will conduct data linkage on a regular basis to access data that is routinely collected by various government bodies, such as data on medication and hospital and aged care utilization. To facilitate this, the ADNeT CQR has collaborated with the Registry of Senior Australians (ROSA) and explored the feasibility of post-diagnostic data collection for the registry using ROSA datasets [15].

The ADNeT CQR published its second Annual Report in May 2023 [16]. At that time, the ADNeT CQR had recruited 55 clinics which were actively contributing data to the registry. Of the 2369 patients captured, 68% (*n* = 1,617) had a dementia diagnosis (median age = 79 years, median Mini-Mental State Exam Score [MMSE] = 22) and 32% (*n* = 752) with MCI (median age = 77 years, median MMSE Score = 27). More than half (53%) of the participants were female, 37% were born overseas, and 27% lived alone. Most participants were independent in activities of daily life and 63% had their first appointment within 90 days of referral. Importantly, this report provided data on Clinical Quality Indicators, which are specifically defined measures that examine the quality of and variations in dementia diagnostic practices and initial management. Of the seven Clinical Quality Indicators, the most variations were evident for appointment wait times and for prescription or recommendation of acetylcholinesterase inhibitors for mild to moderate Alzheimer’s disease.

To drive quality improvement initiatives at participating sites, the ADNeT CQR provides it’s participating sites with individual site reports on a six-monthly basis. These reports include benchmarked data on site participant demographic and clinical profile, site clinical processes, and site Clinical Quality Indicators. They help participating sites to assess their performance compared to their peers and to identify areas for quality improvement at a site level.

The ADNeT CQR is well positioned to support registry-nested trials and other research sub-studies and has a secondary aim to facilitate the recruitment of participants into research and establish a resource available to all to assist further study into the risk factors for, and trajectory of, dementia and MCI in Australia. As amyloid lowering therapies become available, the ADNeT CQR has great potential for the post-market surveillance of the benefits and risks of new medications.

### The ADNeT Memory Clinics Initiative

The MCs Initiative strives to improve access, quality, and equity of care for all Australians who are seeking assessment of their cognition and/or management of their condition. It has, for the first time, established a collaborative network of clinicians providing a framework for the operation of MCs. This includes harmonization of diagnostic and post-diagnostic support pathways to ensure that all Australians have equitable access to high-quality dementia assessments, regardless of location.

The MCs Initiative conducted two initial surveys characterizing the health services of MCs across Australia [5]. Both surveys revealed considerable heterogeneity with core elements of clinical assessment, patient management and service operation [5, 6]. The initial survey [5] nonetheless showed that some neuropsychological instruments were commonly utilized (see https://www.australiandementianetwork.org.au/initiatives/memory-clinics-network/clinical-resources-for-clinicians/) and these have been embedded into a novel automated neuropsychological norming tool (ANNT). Developed in collaboration with the Commonwealth Scientific and Industrial Research Organisation (CSIRO), this free tool automatically calculates normative data for a range of neuropsychological tests and produces a data sheet for clinical or research purposes. Its use in MCs aims to facilitate greater harmonization of neuropsychological assessments between clinics, and ease the time burden for neuropsychologists. Clinicians can access the ANNT here (https://www.australiandementianetwork.org.au/initiatives/memory-clinics-network/annt-registration-form/).

The second survey of 60 MCs conducted in 2020 showed that in comparison to similar countries (e.g., United Kingdom, Ireland, and Canada) the wait-times for Australian clinics were long. Only 28.3% of clinics surveyed were able to offer an appointment within 1–2 weeks for urgent referrals, with significantly more private clinics (58.3%) compared to public clinics (19.5%) being able to do so. For non-urgent referrals, a minority (23.8%) of publicly funded clinics had wait-times of less than 2 months. In general, private clinics were able to offer substantially shorter wait times for initial appointments. However, it is noted that the overall time for an assessment itself may take longer in the private sector due to additional wait times for other investigations, such as neuropsychological services, medical tests, or neuroimaging. Further work is required to determine if the overall time to diagnosis differs in private settings. In terms of post-diagnostic support, survey results showed that these were not commonly provided. For example, only 14.5% and 9.1% provided at least two sessions of cognitive interventions or multidisciplinary reablement packages (often provided in clinical research centers), respectively. Overall, the survey findings, coupled with the imminent rise in dementia and MCI cases, and prospect of amyloid lowering therapies [17], highlight the need for improvement and expansion of Australian MCs to improve timely diagnosis and provide quality post-diagnosticsupport.

The MCs Initiative later in 2020 used a modified Delphi process to hold national stakeholder workshops, which included those with living experience of dementia as well as with clinicians and other stakeholders, to help co-design new national service standards. In November 2021, the first national *Memory and Cognition Clinic Guidelines* for MC services were published, based on the overarching principles of person-centered care, equity, and respect [18]. The Guidelines provide standards for the assessment and post-diagnostic support and care that should be ideally provided by MCs. These are now available from the ADNeT website (https://bit.ly/ADNeTMemoryandCognitionClinicGuidelines). A key recommendation derived from the Delphi process which were adopted into guidelines included the need for MCs to provide an appointment within 30 and 90 days for high-priority (e.g., for prospective clients exhibiting rapid cognitive decline, safety concerns and/or suspected self-neglect or abuse) and routine appointments (i.e., in the absence of high-priority criteria), respectively. Moreover, other recommendations included the need to provide written information at all stages of the diagnostic process including assessment procedures and care plan recommendations, and to provide at least one feedback and follow-up evaluation (ideally within 6–12 months). It is also recommended that MCs should have established post-diagnostic referral pathways and should utilize evidence-based interventions.

The MC Initiative is in regular communication with national and state-based health agencies to advocate for increased staffing and funding to enable MCs to increasingly meet these minimal service guidelines. The MCs Initiative will also develop a national accreditation system to support the Guidelines, including training and evaluation processes, with the majority of quantitative data capture being conducted by the CQR. The Guidelines will also be revised in 2023 to include specific sections for those from culturally and linguistically diverse backgrounds (currently 25% of Australia’s dementia population) [18], as well as for those with intellectual disability.

**Fig. 2:**
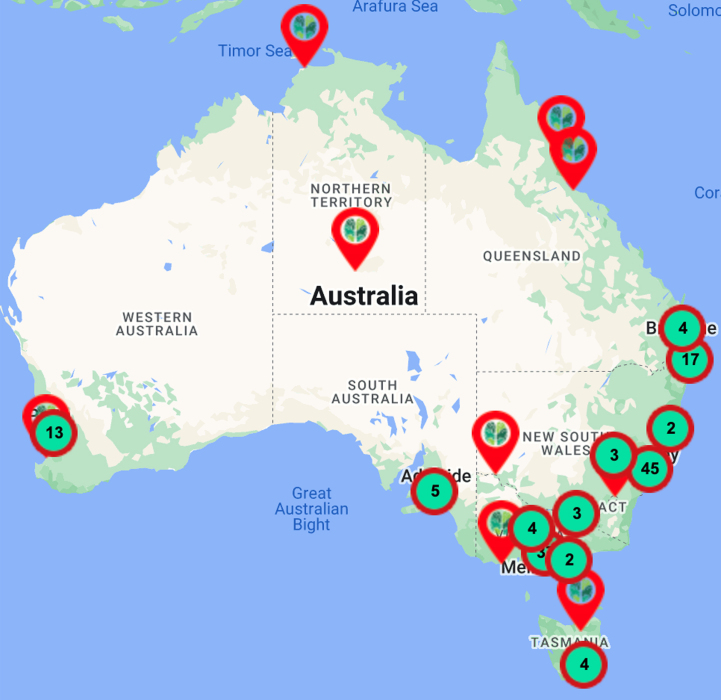
Memory and cognition services (*n* = 149) mapped across Australia as of November 2022.

The MCs Initiative has focused on service and quality improvements and has established bimonthly webinars for health professionals (>3000 registrants across 13 webinars to date, Table 1), and these are also freely available on YouTube via the ADNeT website (https://www.australiandementianetwork.org.au/initiatives/memory-clinics-network/peer-support-for-clinicians/). Concurrently, the MCs Initiative mapped national services of multidisciplinary public, private and hybrid (i.e., clinics embedded within universities, funded via grants with additional use of Medicare) sector MCs for use by general practitioners, the general public, and researchers. Details of the 149 services mapped to date can be accessed from the ADNeT website (https://www.australiandementianetwork.org.au/initiatives/memory-clinics-network/find-a-clinic-or-service/) and have been illustrated in Fig. 2. Moreover, in Table 2 we have extrapolated on the number of older adults ≥65 years that would be expected to undergo an assessment across our mapped network of public multidisciplinary MCs within each state and territory across Australia, revealing that there are significant gaps in the current Australian MC capacity for people with dementia. If cases of MCI and subjective cognitive complaints were to be factored in, the service gap would be substantially greater.

**Table 1:** List of all topics covered by expert presenters over ADNeT-*Memory Clinics* bimonthly webinars series between September 2020 and October 2022

Webinar Number	Topic Name	Number of Registrations
1	ADNeT Screening for Trials and Memory Clinics	212
2	Frontotemporal dementia and Lewy body dementia: Review of current understanding, management and clinical trials	278
3	Development and Function of the Cognitive Dementia and Memory Service (CDAMS)	142
4	Cognitive Interventions for Older Adults: Evidence and a Move Towards Implementation	175
5	Clinical Quality Registries for Dementia and Mild Cognitive Impairment in Australia	91
6	Sleep Disturbance and Dementia	351
7	Post-diagnostic support – how ‘Forward with Dementia’ and Dementia Australia can help	252
8	The Australian Dementia Network – Our Progress to Date and Future Plans	148
9	Vascular Cognitive Disorders	336
10	Keeping Dementia Prevention &Care with Aboriginal &Torres Strait Islander Peoples ‘On TRACK’	182
11	Can Exercise and Nutrition Promote Cognitive Resilience and Reduce the risk of Dementia?	300
12	Interventions Addressing the Behavioural and Psychological Symptoms of Dementia	313
13	Amyloid PET in clinical practice: Interpretation and clinical significance	236

All webinar films can be watched on YouTube via the ADNeT website (https://www.australiandementianetwork.org.au/initiatives/memory-clinics-network/peer-support-for-clinicians/).

**Table 2:** Number of mapped publicly funded MCs in each Australian state that offer a multidisciplinary service (*n* = 54), estimated MCI cases aged over 65 years and dementia cases and unmet need of public memory clinics

	Mapped public MCs, number^A^	Population ≥65 years, number (‘000)^B^	Proportion of population ≥65 years, % ^B^	Estimated new MCI cases/year^C^	Estimated new dementia cases/year^D^	Estimated new MCI+dementia cases/year^C,D,^^E^	Potential MCI+dementia capacity, cases MC, number^C,D^	Estimated total state capacity, per year^F^	Proportion serviced at current capacity
Victoria	22	1,072.6	16	42,904	18,020	60,924	2,769	4,752	7.8%
New South Wales	17	1,393.4	17	55,736	23,409	79,145	4,656	3,672	4.6%
Queensland	7	864.4	16	34,576	14,522	49,098	7,014	1,512	3.1%
Western Australia	3	431.9	16	17,276	7,256	24,532	8,177	648	2.6%
South Australia	4	352.5	19	14,100	5,922	20,022	5,006	864	4.3%
Tasmania	0	116.0	20	4,640	1,949	6,589	NA	0	0%
Northern Territory	1	21.6	9	864	363	1,227	1,227	216	17.6% ^G^
Australian Capital Territory	0	60.5	13	2,420	1,016	3,436	NA	0	0%

MCs, Memory Clinics; MCI, mild cognitive impairment; NA, not assessable; ^A^Number includes public multidisciplinary clinics only and does not include 32 private or research-focused clinics: New South Wales (*n* = 12); Victoria (*n* = 9); Queensland (*n* = 5); Western Australia (*n* = 4); Tasmania (*n* = 2); ^B^Australian Bureau of Statistics, National, State and Territory Population, March 2022; ^C^MCI incidence varies widely, being up to 10.5% (95% confidence interval: 8.16–12.77%) in an Australian sample [32] and between 2.25%–6.01% in international samples [33]. A lower incidence rate of 4% has been used in accordance with Ritchie (2004), assuming not all MCI cases are help seeking for specialist assessment; ^D^Based on an estimated dementia incidence rate of 1.68% in older adults aged ≥65 years as reported by the 45 and Up Study; Welberry, et al. [35]; ^E^Projections of younger onset cases or MCI diagnosed under age 65 years not included in these estimates; ^F^Assumes a total 216 patients/year seen at each public MC based on prior data indicating public clinics assess an average of 3.1 new patients/ day, and run 1.45 clinics /weeks (45% of public clinics provide >1 clinics/week) for 48 weeks of the year [5]. It is noted that some MCs, particularly in regional areas operate only once per month, with substantially less capacity; ^G^Northern Territory clinic has limited multidisciplinary support. Case projections do not consider Aboriginal and Torres Strait Islander population with younger dementia onsets.

In 2024, the MCs Initiative will commence a key feasibility study evaluating the innovative blood-based biomarkers for the detection of Alzheimer’s disease. In partnership with the University of Sydney’s Healthy Brain Ageing Program (Sydney), The Austin Hospital (Melbourne), and the University of Tasmania’s ISLAND Clinic (Hobart), researchers will examine the diagnostic and clinical management impact of a novel blood-based biomarker that can specifically and sensitively detect a phosphorylated tau protein variant in plasma (i.e., pTau181) [19] (ANZCTR Identifier: ACTRN12622000515796). It is hoped that in the future, the blood test will improve diagnostic accuracy of Alzheimer’s disease and will be more cost effective and accessible than the current invasive, costly and specialist methods available now for gold-standard diagnosis, namely the use of PET and CSF testing. Indeed, the MC national survey showed that 96.2% of clinicians would utilize blood-based biomarkers in their clinical practice if they were available [20]. The development and broader scale implementation of the biomarkers would enable a faster and more accurate screening for Alzheimer’s disease. In turn, this will assist with more accurate and faster diagnoses, and facilitate access to appropriate and targeted amyloid lowering or secondary prevention treatments. In order to close the evidence to practice gap in post-diagnostic support for people with MCI and dementia, the MCs Initiative has commenced a feasibility study to implement cognitive interventions for people with MCI in MCs. Cognitive interventions have a robust evidence-base for improving cognition using both strategy and computerized interventions [21–23], with a recent meta-analysis of 26 studies demonstrating that individuals with MCI who received multicomponent training or interventions targeting multiple domains (including lifestyle changes) displayed an improvement on outcome measures of cognition post-intervention [24]. In addition, the American Academy of Neurology recommends that cognitive training should be offered in MCI [25]. Despite this robust evidence base, few clinics are able to offer such interventions to their patients. The working party (for more information, visit: https://www.australiandementianetwork.org.au/initiatives/memory-clinics-network/cognitive-intervention-working-group/) has undertaken a scoping review and workforce survey; and in 2023, provided training to neuropsychologists ahead of a formal implementation trial across five Australian states. Key outcomes will include workforce and service readiness, enablers and barriers to effective implementation and process and contextual factors influencing uptake.

### The Screening for Trials Initiative

The ADNeT STI seeks to accelerate the development of effective therapies to prevent or treat dementia and give more Australians access to the latest potential therapies through participation in trials. The Initiative has leveraged and expanded major seminal studies in the field, including the Australian Imaging Biomarkers and Lifestyle (AIBL) study, and has engaged closely with international leaders in clinical trials, including industry-sponsored trials to encourage large trials to be brought to Australia. Many of the trials are predicated on the discovery that the slow build-up of amyloid-β (Aβ) and tau are key to the pathophysiology and underpin the clinical symptoms of Alzheimer’s disease. Notably, these begin to deposit in the brain 15 to 20 years before other clinical symptoms develop [26, 27] and can be detected using PET or CSF biomarkers.

The ADNeT STI has now established key screening sites in all mainland states that provide a comprehensive assessment of persons who are interested in participation in clinical trials. This includes persons with mild dementia, MCI, and cognitively normal individuals at an increased risk of developing Alzheimer’s disease. After an initial phone screen, those who may be suitable for a clinical trial undergo neuropsychological testing, structural magnetic resonance imaging (MRI) and amyloid PET scans as well as some also receiving a tau PET scan. The findings are discussed with the participant and their doctor. Currently recruiting trials are also discussed, and those that choose to participate in a clinical trial are referred to the relevant trial site. This service is provided in Victoria by the Florey Institute of Neuroscience and Mental Health with Austin Health and the University of Melbourne; in New South Wales by Macquarie University, and The University of Newcastle; in South Australia by the South Australian Health and Medical Research Institute with the Royal Adelaide Hospital; in Western Australia by the Alzheimer’s Australia Research Foundation with Edith Cowan University and the Sir Charles Gardner Hospital; and in Queensland by the University of Queensland with the Royal Brisbane Hospital and the Queensland Institute of Medical Research. The trial sites, wherever possible, utilize standardized protocols and tracers including NAV4694 for Aβ and MK6240 for tau PET scans [28, 29]. To support trial recruitment, STI has established a dedicated Volunteer Portal (https://www.australiandementianetwork.org.au/research-volunteer-portal/) that screens volunteers from the community and directs them to appropriate ADNeT centers for screening and if appropriate, available trials are offered. As of January 2023, ADNeT STI has completed 1,022 screens (mean age = 68.5 years; female, 52%; formal years of education = 13.3 years), with all participants having undergone Aβ PET imaging (*n* = 1,022/1,022, 100%), in addition to an MRI scan (*n* = 1022/1022, 100%), blood work-up (*n* = 951/1,022, 93%) and tau PET (*n* = 225/1,022, 22%). To date, all participants have completed neuropsychological assessment, with 588 participants (57%) having undergone basic assessment and 434 participants (43%) having undergone a full assessment.

Although impacted by government mandated COVID-19 lockdowns and general disruption throughout 2021–2022, the ADNeT STI has recruited and moreover referred over 1,000 fully evaluated individuals to ADNeT affiliated clinical trial sites. It has recruited 3,353 registrants into the ADNeT Volunteer Portal as of January 2023 (mean age = 67.5 years; male, 60%). It has attracted further grant funding from international pharmaceutical companies and collaborates with other national cohorts (e.g., Prospective Imaging Study of Ageing [PISA]) and consortia (e.g., NHMRC Centres of Research Excellence) to synergize research efforts. It has also introduced blood biomarker (i.e., pTau181) and genetic testing for the Apolipoprotein E *ɛ*4 allele (*APOE* *ɛ*4) to assist in the detection of very early Alzheimer’s disease and as a pre-screen before conducting PET scans. ADNeT has also facilitated production of Aβ and tau PET tracers in each mainland state. This is not only vital for clinical trials but will also be crucial in providing Australians access to the necessary tests required to access emerging treatments for Alzheimer’s disease.

### Support initiatives, stakeholder engagement, and partnerships

To support data platforms, quality, and data analytics, ADNeT partners with the CSIRO who bring expertise in information technology, data science and big data analytics (e.g., machine learning and artificial intelligence). It has recently expanded its partners to include the National Ageing Research Institute (NARI) who bring complementary expertise in aged care and translational research in the care sector.

As a whole, ADNeT has forged close connections and cross-collaborations within the Australian research community. For example, with national NHMRC Centres of Research Excellence focused on diagnostics (led by Prof. Scott Ayton), vascular dementia (led by Prof. Perminder Sachdev), sleep disturbance (led by Prof. Sharon Naismith), and indigenous populations (led by Prof. Dina LoGiudice). It also has close synergies with novel investigator-led lifestyle trials for dementia prevention such as the Australian AU-ARROW trial (led by Prof. Ralph Martins), which is part of the WORLDWIDE FINGERS network [30], and a Medical Research Future Fund program of blood-based biomarker development and implementation leverages the strengths and capabilities of ADNeT (led by Prof. Ashley Bush and Prof. Christopher Rowe). ADNeT works closely with the peak people with living experience body, Dementia Australia, and people with living experience and/or their carers are core members of each Initiative’s steering committee. In addition, support and hosting of the Early and Mid Career researcher Young Onset Dementia Interest Groups’ initiatives enables knowledge exchange and cross-talk.

In addition, ADNeT has regular information exchange and strategy discussions with the Department of Health, state health departments and the Australian Institute of Health and Welfare to advise on issues regarding health service improvements, national data quality improvements and other national dementia plans. The MCs Initiative regularly engages with Commonwealth and state health departments to highlight issues pertaining to expansion and funding of national dementia assessment and treatment services, particularly in key areas of need such as in regional, rural, and remote areas of Australia. The MCs Initiative is also collaborating with the AIHW and Dementia Australia to obtain new data on timely diagnosis and national disparities in healthcare provision and out-of-pocket costs.

ADNeT investigators work closely with the pharmaceutical industry and other industry partners to inform on research, workforce and health service issues as well as advising on training strategies and opportunities. In particular, ADNeT will work closely with industry regarding the implementation of plasma biomarkers, and facilitating access to, and the monitoring of amyloid lowering therapies (e.g., monoclonal antibodies).

For governance and sustainability, ADNeT has established an effective governance structure. Governed by a multidisciplinary Management Committee comprising Chief/Principal Investigators from participating institutions, ADNeT works closely and in partnership with major people with living experience advocacy groups such as Dementia Australia and Alzheimer’s Western Australia and engages with state and federal Government bodies, people living with dementia and their carers.

### Australian dementia network limited (ADNL)

In 2020, the Australian Dementia Network Limited (ADNL) was established. This enables ADNeT to easily enter into contracts with the pharmaceutical industry, philanthropy and government agencies. ADNL is incorporated as a not-for-profit company with charitable status to facilitate industry investment and donations, attract additional income (e.g., from state governments, funding bodies or philanthropy) and in turn enable ADNeT to be able to operate beyond the life of the research grant. In principle, ADNL could coordinate dementia research funding and development throughout Australia and assist in setting strategic directions for Australia’s future dementia research, workforce, and infrastructure needs.

To date, ADNL has been the conduit for over $AUD 7 million investment from the pharmaceutical industry, through the support of participant screening through ADNeT to facilitate entry into clinical trials, for provision of samples and data—often in partnership with AIBL and for the development of the CQR to enable post-marketing surveillance.

### National and international leadership

ADNeT has established a leading presence in the Australian health and research community and now leads the Australia Dementia Research Forum (from 2021 onwards), a major Australian dementia conference. It also supports the Early and Mid-Career Researchers ‘Accelerator Group’ and via Steering Committees of the CQR and MCs Initiatives, it encompasses a diverse range of stakeholders. ADNeT has furthermore forged links with global dementia research organizations in the United Kingdom, United States and in Europe.

## DISCUSSION AND FUTURE DIRECTIONS

Over the last four years, ADNeT has innovated in the fields of diagnostics, quality and harmonization of healthcare, clinical trials, clinical care professional development, and has commenced translational research into key healthcare initiatives such as cognitive training and the implementation of biomarkers into clinical practice. The capacity building, knowledge transfer and healthcare network initiatives have been equally successful and have required significant investment and national cooperation.

Future directions for the network and field include the need to support primary care in dementia diagnostics and dementia prevention efforts, to expand health services and address inequalities in care provision in regional and rural areas, to ensure provision of gold-standard diagnostics and interventions and to bolster post-diagnostic support. The CQR is now well positioned to include a national case registry for amyloid lowering therapies as well as data linkage of our CQR data with national health datasets (e.g., Medical Benefit Schedule, and Pharmaceutical Benefits Scheme data linkage). The MCs Initiative will strive to improve harmonization, quality and equity of services and will work closely with the CQR on memory clinic accreditation. The MCs and STI Initiatives will work collaboratively on implementing new innovations in diagnostics and treatments and in partnership with government, health services and industry.

In addition, ADNeT will continue to emphasize research enablers, capacity building, and research translation within the field. It will continue its inclusive work with Early and Mid-Career Researchers, and to coordinate Australia’s premier dementia research conference, The Australian Dementia Research Forum. The future vision will include new initiatives such as:

• **Health services and policy:** ADNeT will seek new opportunities to build the skills and confidence of GPs to diagnose and treat MCI and dementia, and to implement secondary prevention strategies for risk reduction. ADNeT will undertake a coordinated approach on early detection, treatment, and prevention of dementia in primary care by:
• Implementing a new, affordable, and accurate blood test for diagnosis of Alzheimer’s disease;
• Upskilling GPs on early diagnosis (blood test, streamlined cognitive test, and CT scan) and empowering primary health network with team-based dementia diagnosis and care;
• Implementing new clinic models to boost health system capacity (virtual clinics in regional areas);
• Clinical trials of newly approved amyloid lowering therapies (DMT), including tracking and monitoring uptake, appropriate use, safety, and benefits of the amyloid-lowering therapies required for post marketing surveillance, and;
• Facilitating evidence-based lifestyle interventions (exercise, cognitive training, sleep, diet) to those at risk of dementia.

Exciting research breakthroughs in diagnostics and treatment for Alzheimer’s disease, that accounts for 70% of dementia, have been recently achieved. Recent developments in early detection, disease modifying therapies, risk factor reduction approaches, and non-drug post diagnostic intervention now make reduction in the prevalence of dementia in Australia feasible. Recent breakthroughs in amyloid lowering agents for Alzheimer’s disease such as Lecenemab and Donanemab necessitate a radical change in our approach to diagnosis and treatment and presents major challenges to the Australian health care system. Strengthening primary health care through multidisciplinary team-based care models, significant up-skilling of GPs, specialists and new quality assurance mechanisms are needed. Early diagnosis is critical for maximal benefit from disease modifying agents. Research collaboration is key to achieve results and economies ofscale.

• **Expansion of the network:** The current focus on Alzheimer’s disease will be expanded to include the less common forms of dementia such as vascular dementia, dementia with Lewy bodies, and frontotemporal dementia.
• **Collaboration in clinical trials:** Collaboration with international pharmaceutical companies and Australian academic researchers will support clinical trials of new therapeutics, lifestyle trials and new trials of health service improvements.
• **Facilitation of Australia’s dementia biomarker informed research**. ADNeT will foster internationally competitive research by providing highly characterized individuals at risk of, or living with dementia, and enabling ethically approved access to their valuable cognitive and lifestyle data, blood and CSF biospecimens, MRI and PET imaging data, and details on disease trajectory and health outcomes.
• **Capacity building:** ADNeT will play a national leadership role in building of the workforce across multidisciplinary areas of dementia research, including seeking new funding opportunities to support the workforce, foster impactful research and in key areas such as translational research and media and community dissemination.
• **Industry engagement:** Through ADNL as a granting body, attract and support the brightest minds and new ideas for prevention and therapy to capitalize on recent advances in monoclonal and other immunological therapies and the dramatic advances occurring in genetic based therapy through mRNA modulators.

The COVID-19 pandemic has demonstrated the need for greater and more proactive planning when it comes to population health issues. For diseases with high probability and low visibility such as dementia, the argument for adequate investment in research and development efforts has become even more compelling. Clearly, there is a need for diversity of research, from population health and dementia prevention to health services research across the spectrum of primary care, MCs, community and aged care to basic science and biobanking. Integration of new technologies for diagnostics, tracking and interventions also warrants consideration, and could be cost effective and scalable widely, if designed and/or adapted with the end-user and health system in mind. In this regard, co-design, inclusion of living experience experts and implementation science methods should be integrated into future research initiatives that are seeking research translation and health impact. Expansion of the network to clearly identify non-Alzheimer’s dementia (e.g., frontotemporal dementia) will be a key goal. In line with other national research [31] there is a need for greater inclusion of those from culturally and linguistically diverse communities, and for specific initiatives focused on Aboriginal and Torres Strait Islander peoples. In addition to core research, there is a continual need to emphasize research enablers, capacity building, and research translation within the field and to support and bolster Australia’s Early and Mid-Career Researchers.

Overall, over the past four years ADNeT has demonstrated the benefits and successes of a nationally coordinated research effort engaging with industry, health policy makers, people with living experience and key partner organizations. Coupled with ongoing global developments in diagnostics, plasma biomarkers, effective amyloid lowering therapy, management and post-diagnostic care – we have laid the foundations to springboard from these efforts in order to expand and up-scale ADNeT, and ultimately to optimize the impact on the individual at risk of, or living with dementia alongside their support network.

## Data Availability

Data sharing is not applicable to this article as no datasets were generated or analyzed during this study.
